# Prevalence of *Toxoplasma gondii* antibodies, circulating antigens and DNA in stray cats in Shanghai, China

**DOI:** 10.1186/1756-3305-5-190

**Published:** 2012-09-05

**Authors:** Quan Wang, Wei Jiang, Yong-Jun Chen, Chun-Ying Liu, Jin-lei Shi, Xin-tong Li

**Affiliations:** 1 Department of Veterinary Public Health. Chinese Academy of Agricultural Sciences CAAS, Shanghai Veterinary Research Institute, 518 Ziyue Road, Minhang, Shanghai 200241, PR China

**Keywords:** Prevalence, *Toxoplasma gondii*, Stray cats, Ab-ELISA, CA-ELISA, PCR, Shanghai, Epidemiology, Environment, Indicator

## Abstract

**Background:**

*Toxoplasma gondii* is prevalent in most areas of the world and may cause abortions or neonatal complications in humans. As the only definitive host, cats play an important role in the epidemiology of the disease. Infection rates in cats, especially stray or free-living cats, are considered to be the best sentinels of the level of *T. gondii* in the environment. The *T. gondii* infection can be diagnosed in different ways with different methods depending on the target. However, little information on *T. gondii* infection in cats was available in Shanghai, China. Moreover reports on prevalence of circulating antigens, antibodies and DNA of *T. gondii* in the same study are rare.

**Methods:**

In the present study, the presence of antibodies (Ab), circulating antigens (CA), and/or DNA of *Toxoplasma gondii* in samples from 145 stray or unwanted cats from 6 animal shelters in Shanghai (China) was determined in order to estimate the prevalence of *T. gondii* infection, by Ab-ELISA, CA-ELISA, and nested-PCR, respectively.

**Results:**

The positive rates for the antibodies, circulating antigen and DNA of *T. gondii* were 11.7% (17 of 145), 5.5% (8 of 145) and 5.71% (2 of 35), respectively*.* No cat tested was positive by both the Ab-ELISA and the CA-ELISA, but the results of the PCR were consistent with the CA-ELISA assay. Therefore, the overall estimated prevalence of toxoplasmosis was 17.2% (25 of 145). According to our results, the positive rates of specific antibodies and circulating antigen of *T. gondii* were significantly different between adult cats (>1 year old) and juvenile cats (≤1 year old); the former was 13.5% versus 3.9% by Ab-ELISA, while the latter was 1.7% versus 23.1% by CA-ELISA. From the results obtained with all three detection methods used in this study, the rate of infection was not significantly different between male and female cats (P ≥0.05); and the overall rate was 17.9% for males versus 16.4% for females.

**Conclusions:**

The results suggest that detection of circulating antigens (CA) is necessary in surveys of *T. gondii* infection, especially for juvenile cats. Our investigation revealed that the prevalence of *T. gondii* infection in stray cats in Shanghai is high. Control programs are needed for stray cat populations in order to reduce the risk of zoonotic transmission of toxoplasmosis to other domestic animals and humans, especially females.

## Background

Toxoplasmosis is a worldwide endemic disease caused by *Toxoplasma gondii* infecting a broad spectrum of vertebrate hosts, including humans. *Toxoplasma gondii* infection can cause toxoplasmic encephalitis in immunocompromised patients, blindness, abortion, fetal abnormalities or even prenatal death in congenital cases [1-3]. Cats play an important role in the epidemiology of the disease, as they are the definitive hosts allowing the sexual phase of the parasite in the gastrointestinal tract [4]. Shanghai is the most significant industrial and commercial city in China and is one of the largest metropolitan areas in the world. The size of the cat population has increased significantly with the recent improvement in people’s living standards and awareness of good animal welfare. In comparison with pet cats, stray or free-living cats are especially important to public health, because they are considered to be the best sentinels of the level of *T. gondii* in the environment. This is because they roam freely without any protection from pathogens and they can contract feline toxoplasmosis by predation of infected animals with *T. gondii* cysts: birds, rodents, other wildlife, or by ingesting of undercooked meat from human refuse. Infected cats shed millions of infective oocysts in their feces during a brief period that can contaminate human food and/or water, and may infect other mammals and birds [5].

Infection rates in cats, especially stray or free-living cats, give an indirect indication of the prevalence of *T. gondii* in the environment, but little attention has been focused on this matter and there have been limited surveys of *T. gondii* infections in cats in Shanghai in recent years.

Infection with *T. gondii* can be diagnosed in a number of ways, such as an antibody detection enzyme linked immunosorbent assay (Ab-ELISA) that is based on the detection of specific antibodies in serum samples from hosts, detection of circulating antigens in serum samples using a CA-ELISA, and polymerase chain reaction(PCR)for various target genes. Serological surveys are good indicators of the occurrence of *T. gondii* infection in cats because serologically positive cats probably shed oocysts [6,7]. However, in general, positive detection of immunoglobulin G (IgG) indicates *Toxoplasma* infection but does not give any indication of when the infection occurred. Circulating antigens are easily detected during the acute stage of infection and are considered to provide direct evidence of the presence of an infection. The PCR detection of *T. gondii* DNA from biological samples shows good specificity and sensitivity in the diagnosis of toxoplasmosis [8-10].

Surveys of *T. gondii* infections in cats have been performed in various provinces of China in recent years [11,12]. However, there have been limited recent surveys of *T. gondii* infections in cats in Shanghai. To give an indication of the environmental spread of *T. gondii*, and thus to provide a foundation for the control improvement of the parasite infection in stray cats in Shanghai, China, we propose in our current investigation to estimate the prevalence of *T. gondii* in stray cats . Previous surveys for *T. gondii* infection have been conducted generally by detection of antibodies or DNA, while antigens were not considered. To our knowledge, there are few studies that have involved the detection of circulating antigens of *T. gondii* (TCA) in cats, and only a few reports of the detection of TCA in murine and human toxoplasmosis by the use of ELISA [13,14]. Therefore, in this study, we investigated *T. gondii* infection in stray cats from six animal shelters in Shanghai on the basis of *T. gondii* circulating antigens (TCA), *T. gondii* antibodies (TAb) and DNA using CA-ELISA, Ab-ELISA, and nested-PCR, respectively.

## Methods

### Animals

Between January, 2010 and December, 2011, a total of 145 unwanted and stray cats were selected randomly from six animal shelters, which receive unwanted pet cats offered for adoption by their owners, and stray cats from the streets or residential areas of the city of Shanghai, China. General data on these cats, including age, gender, and health status, were obtained from the owners of the animal shelters, or were estimated on the basis of body condition and examination of dentition. To reduce the chances of cross-contamination with other cats, the stray cats were isolated individually in a cage when they were first adopted and their feces were cleaned up daily; they remained in good health. All the cats were asymptomatic; moreover, none had received any prophylaxis or treatment for *T. gondii*. This study was approved by the Animal Ethics Committee of Shanghai Veterinary Research Institute, Chinese Academy of Agricultural Sciences (No. SYXK<HU>2011-0116).

### Blood samples and serum or DNA preparation

Blood samples were collected from the saphenous veins of the cats into plain sterile tubes, left to clot at room temperature for 3 h, and centrifuged subsequently at 1,000 *g* for 10 min. The separated sera were stored at −20°C until analysis. In addition, 35 blood samples (from 8 juveniles and 27 adults) chosen randomly from these cats were placed into tubes containing heparin for the analysis of *T. gondii* DNA by nested PCR.

### Determination of antibodies to *T. gondii*

All 145 serum samples were tested for antibodies to *T. gondii* using a commercial ELISA-CIVTEST® kit (Combined Company, Shenzhen, China) according to the manufacturer’s recommendations. The manufacturer’s test report showed that the sensitivity and specificity of the Ab-ELISA kit were 95% and 100%, respectively. Positive and negative control sera were provided in the kit, and the dilution medium was used as the blank control. Briefly, the *T. gondii* specific antigen was used to coat a 96-well ELISA plate. After incubation of a diluted serum sample (1:100) in the test well and subsequent washing, a conjugate was added. The plate was washed again, and a chromogenic enzyme substrate was added. Finally, the optical density (OD) value at 450 nm was recorded using a photometer (BioTek, Gene Company Limited, USA). All the values were recorded after appropriate blank correction. A serum sample was considered positive for *T. gondii* if the value was 2.1 times higher than that of the negative control sample. Each sample was tested in duplicate on two repeat assays.

### Determination of circulating antigen of *T. gondii*

Circulating antigens of *T. gondii* were investigated in all serum samples using a commercial Circulating Antigen Detection ELISA (CA-ELISA) kit (Combined Company, Shenzhen, China). The manufacturer’s test report showed that the sensitivity and specificity of the CA-ELISA kit were 97% and 100%, respectively. Positive and negative control sera were provided in the kit. The procedures described in the manufacturer’s instructions were followed carefully. Briefly, the plates were shaken gently for 2 min and incubated at 37°C for 2 h without shaking. Diluted washing liquid (40 μl) was added to each test well, and mixed with 100 μl of each serum sample. At the same time, we set the negative, positive and blank control groups, to which were added 50 μl of the negative and positive control samples and the diluted washing solution, respectively. The samples were mixed with 50 μl diluted enzyme conjugate (except the blank well) at 37°C for 60 min. The liquid in the well was discarded; the wells were washed with the diluted washing liquid five times at intervals of 1 min, and the wells were tapped dry. Substrate and color developing reagent were added to each well successively before incubation at 37°C for 10 min in the dark. Subsequently, the stop buffer was added to each well to terminate the reaction. The OD value was read by a photometer (BioTek, Gene Company Limited, USA) at 450 nm; the zero setting was obtained from the blank control group. A sample was considered positive for *T. gondii* if the value was 2.1 times higher than that of the negative control sample.

### Detection of *T. gondii* DNA

Thirty-five blood samples were chosen randomly and the DNA was extracted using a DNA rapid extraction kit (UltraPure, Shanghai, China). Detection of *T. gondii* was carried out by nested PCR amplification of the 5.8S rRNA gene as described in our previous report [15], in which two pairs of oligonucleotide primers directed against the 5.8S rRNA gene (Accession No. X75453) of *T. gondii* were used. The outer primers were 5'-ACCTTTGAATCCCAAGCA-3' and 5'-TTTGCATTCAAGAAGCGTG-3', and the inner primers 5'-TAAATCGGACAAACGCCC-3' and 5'-AAGGTGCCATTTGCGTTC-3', which should amplify about a 433-bp fragment under the predetermined conditions. The PCR mixture for each reaction contained 2 μl of the extracted DNA sample, 19 μl double-distilled water, 2 μl of each specific outer primer (each 10 μM), 25 μl of the 2 × PCR mix (Dongsheng Biotech, Guangdong, China), which consisted of 100 mM KCl, 20 mM Tris–HCl, 3 mM MgCl_2_, 400 μM dNTPs, and 0.1U/μl Taq DNA polymerase. Amplification was performed using an Authorized Thermal Cycler (Eppendorf, Germany) with an initial denaturation step of 94°C for 4 min, followed by 30 cycles of 30 s at 94°C, 45 s at 55°C, and 1 min at 72°C. Subsequently, 1 μl of the first PCR products were transferred into a second tube containing 49 μl of the reaction buffer, as described in the first round reaction, for the second round of the same 30 cycles of amplification, but with the pair of inner primers. A positive control containing purified DNA equivalent to five *T. gondii* tachyzoites was included. A negative control containing no DNA was also included, by adding equivalent volumes of double-distilled water. The PCR products of the positive samples were analyzed by electrophoresis using 2% TBE agarose gels and were extracted from the gel using the AxyPerp^TM^ DNA Gel Extraction kit (Axygen, Union City, USA). They were cloned into the pMD®18-T Simple vector (TaKaRa, Dalian, China), and sequenced by Shanghai Invitrogen Biotechnology Company.

### Statistical analysis

Statistical analysis of differences in the prevalence of *T. gondii* between the juvenile and adult cats, and between male and female cats, was performed using the Chi Square Test program in SPSS for Windows (Release 16.0 standard version, SPSS Inc., Chicago, America) and Excel 2003 (Microsoft®). The differences were considered statistically significant when P <0.05.

## Results and discussion

In total, 145 unwanted and stray cats (78 male, 67 female) were examined both by Ab-ELISA and CA-ELISA. Of these, 26 were juveniles (≤1 year old) and 119 were adults (>1 year old). As shown in Table 1, the positive rates for antibodies, circulating antigen and DNA of *T. gondii* were 11.7% (17 of 145), 5.5% (8 of 145) and 5.7% (2 of 35), respectively*.* The results of the PCR were in agreement with those of the CA-ELISA assay, but no cat tested positive both by the Ab-ELISA and the CA-ELISA. When the results of the CA-ELISA and Ab-ELISA were combined, the total estimated prevalence of toxoplasmosis was 17.2% (25 of 145), which was lower than that reported in other countries [16,17] and in Guangzhou, Beijing and Hebei provinces in China [11,18,19]. 

**Table 1:** **Prevalence of antibodies, circulating antigen and DNA of* T. gondii *in stray cats by gender and age using ELISA (Ab), ELISA (CAg) and Nested-PCR**

	**ELISA(Ab)**			**ELISA(CAg)**			**Nested-PCR**			**Total cats**		
	**Total**	**Positive**		**Total**	**Positive**		**Total**	**Positive**		**Total**	**Positive**	
**Cat group**	**No.**	**No**	**%**	**No.**	**No**	**%**	**No.**	**No**	**%**	**No.**	**No**	**%**
Gender												
Male	78	9	11.5	78	5	64	16	1	6.3	78	14	17.9
Female	67	8	11.9	67	3	4.5	19	1	5.3	67	11	16.4
Age (years)												
≤1	26	1	3.9	26	6	23.1	8	2	25	26	7	26.9
>1	119	16	13.5	119	2	1.7	27	0	0	119	18	15.1
Total	145	17	11.7	145	8	5.5	35	2	5.7	145	25	17.2

In this investigation, the results of the Ab-ELISA and CA-ELISA suggested that the positive rate of *T. gondii* infection varied with respect to age (adults versus juveniles), but the results were different. The prevalence of antibody was in accordance with other studies, where a higher prevalence was observed in adults compared with juvenile cats [11,20,21]. When calculated on the basis of specific IgG antibodies against *T. gondii* via Ab-ELISA, the prevalence of infection in adult cats (13.5%, 16 of 119) was significantly higher (P < 0.05) than that in juveniles (3.9%, 1 of 26), which showed that, with an increase in age, the rate of antibody to *T. gondii* increases. However, in the analysis of circulating antigen to *T. gondii* by CA-ELISA, juvenile cats showed a significantly higher (P < 0.05) frequency of infection (6 of 26, 23.1%) than adult cats (2 of 119, 1.7%). Many studies have shown that circulating antigens are detectable in the sera within a short time period, in the acute phase, in the majority of animals infected with toxoplasmosis. For example, Wang Y H *et al*. [22] found that circulating antigens could be detected from days 2 to 14 post-infection (PI), with a maximum peak of detection on days 4 to 6 PI, in pigs, goats, rabbits and sheep. Similar findings were reported by Chen *et al*. [23], and Bitkowska *et al*. [24].

In the juvenile cats, the prevalence of infection with *T. gondii* as determined by CA-ELISA (23.1%, 6 of 26) was significantly higher (P < 0.05) than that obtained using the Ab-ELISA (3.9%, 1 of 26). In the adult cats, the prevalence of infection with *T. gondii* by CA-ELISA (13.5%, 16 of 119) was also significantly higher (P < 0.05) than that obtained using the Ab-ELISA (1.7%, 2 of 119). Other studies have shown that, with an increase in age, the risk of exposure to *T. gondii* increases [25]. However, in our study, when the results of the CA-ELISA and Ab-ELISA were combined, the total positive rate for the juveniles (26.9%, 7 of 26) was significantly higher (P < 0.05) than that of the adult cats (15.1%, 18 of 119). Taken together, our results suggest that use of the CA-ELISA is necessary to obtain a diagnosis in the acute stage of feline toxoplasmosis, especially in juvenile animals because most juvenile animals, including cats, are likely to be in the acute stage of toxoplasmosis and have not produced sufficient antibodies for detection. In many adult cats, the level of circulating antigens of *T. gondii* has declined below the level of detection, but specific antibodies against *T. gondii* have been produced. Our study showed that juvenile stray cats are commonly infected with *T. gondii* and generally at the acute stage of toxoplasmosis, while the adult stray cats tend to have been infected with *T. gondii* in the past and have already produced antibodies against *T. gondii*.

**Figure 1:**
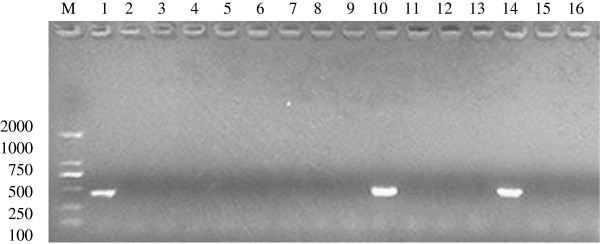
**Detection of 5.8S rRNA gene of* T. gondii *by nest-PCR analysis on agarose gel electrophoresis (1.0%).** The picture show the results of the second round of nest-PCR with the pair of inner primers. Lane M is the molecular weight marker (MW) DL-2000 (TaKaRa Biotechnology Co. Ltd) which showed bands as indicated. Lane 1 is the result of positive control with an about 433-bp fragment; Lanes 2 is the result of negetive control; Lanes 3–9,11-13,15,16 are the results of negetive samples ; Lanes 10 and 14 are the results of positive samples. Data for other 21 cat blood samples not shown but results were the same for negative samples.

In the analysis of the 5.8S rRNA gene of *T. gondii* via nested-PCR, 2 of 35 (5.7%) blood samples, both from juvenile cats, exhibited positive reactions (Figure 1). The positive PCR samples were sequenced, and the DNA sequences were analyzed using BlastN. The result showed that the purified PCR products were 433 bp and 428 bp in length, and the sequence analysis revealed that the cloned gene had 99.8% and 98.4% homology with the corresponding sequence of the *T. gondii* 5.8S rRNA gene from NCBI (Accession No. X75453). Our sequenced products were also submitted to GenBank and the accession numbers are JX456456 and JX456457. The results of the PCR were consistent with the CA-ELISA results because the two PCR-positive blood samples were found to be positive by CA-ELISA, and all the PCR-negative blood samples were negative by CA-ELISA. Some studies have demonstrated that the development of a highly sensitive and specific PCR protocol to identify *T. gondii* DNA can aid the early diagnosis of toxoplasmosis [26]. Van Knapen *et al*. [13] and Hafid *et al.*[14] have confirmed that circulating antigens can be detected in sera from host animals during the acute stage using a sensitive ELISA. In our previous reports, we have shown that our PCR assay is extremely sensitive when used for detection of *T. gondii* DNA in naturally occurring host infections [15,27]. 

In the current study, only 35 of 145 cats were tested by nested-PCR, because sometimes it was hard to collect enough blood samples for all the three methods from the saphenous veins of these cats. As in this study, the Ab-ELISA and CA-ELISA tests were the main methods used to analyze the seroprevalence of *T. gondii* infection in stray cats. So only if the volume of the blood sample collected was excessive for the Ab-ELISA and CA-ELISA tests, then we would put the remaining blood samples into tubes containing heparin for the analysis of *T. gondii* DNA by nested PCR. However, the absolute agreement between the PCR and CA-ELISA suggests that the PCR has a similar specificity to the CA-ELISA, and that both the methods are sensitive and specific and are suitable for use in prevalence studies and diagnosis of the acute stage of feline toxoplasmosis.

Many studies have shown that sex is not considered to be a determining factor for infection with *T. gondii*. Our results also demonstrated that there was no significant difference in the prevalence between the genders (P ≥0.05). The Ab-ELISA assay gave positive results in 11.5% (9 of 78) males and 11.9% (8 of 67) females. The CA-ELISA assay gave positive results in 6.4% (5 of 78) males and 4.5% (3 of 67) females. For the PCR assay, 6.3% (1 of 16) males and 5.3% (1 of 19) females were positive. Combining the results from all three assays gave an overall positive rate of 17.9% (14 of 78) in males and 16.4% (11 of 67) in females. Similar findings were reported by H. Zhang *et al*. [11] in Guangdong in China and Haddadzadeh *et al*. [17] in Iran.

## Conclusions

Free-living animals such as stray cats, dogs, and foxes can be surveyed as indicators of the environmental spread of *T. gondii*[28]. Stray cats are especially important as they shed environmentally resistant oocysts in their feces, and they are increasing in number gradually. Our results suggested that detection of circulating antigens (TCA) is necessary in surveys of *T. gondii* infection, especially in juvenile cats. Our current investigation revealed that the prevalence of *T. gondii* infection in stray cats in Shanghai is high. Given that, many cat owners let their cats play outside allowing contact with abandoned and free-roaming cats, which can lead to ample opportunity for *T. gondii* oocysts to spread in the environment and thus lead to transmission to humans. Human *Toxoplasma* infection may result from direct transmission of the parasite to people from cats [29,30], which are probably the main source of *T. gondii* infection. In order to protect public health more measures should be taken to reduce environmental contamination with *T. gondii* oocysts by stray and unowned cats in Shanghai. Such measures should include the creation of more animal shelters. Free-roaming cats, including animals abandoned by their owners, have be placed in shelters or surrendered to rescue animals where they have an opportunity to be adopted, rather than left to live outside without provisions. In the interim, measures to mitigate the environmental impact of cats, if implemented consistently nationwide, could reduce the extent of soil contamination with *T. gondii* oocysts and the risk to human health.

## Competing interests

The authors declare that they have no competing interests.

## Authors’ contributions

QW and WJ conceived and designed the study, and critically revised the manuscript. QW, WJ, YJC ,YCL,JLS and XTL performed the experiments. WJ analysed the data and drafted the manuscript. All authors read and approved the final manuscript.
